# Supplementary material for: The structure of a dimeric form of SARS-CoV-2 polymerase
Source: Commun Biol. 2021 Aug 24;4:999. doi: 10.1038/s42003-021-02529-9 (PMC8385044; doi:10.1038/s42003-021-02529-9)
Supplement: Supplementary file 2 — Supplementary Information [file 42003_2021_2529_MOESM2_ESM.pdf]

Dimeric form of SARS-CoV-2 polymerase

Florian A. Jochheim[1], Dimitry Tegunov[1], Hauke S. Hillen[2,3], Jana Schmitzová[1], Goran Kokic[1], Christian Dienemann[1], Patrick Cramer[1*]

[1]Max Planck Institute for Biophysical Chemistry, Department of Molecular Biology, Am Fassberg 11, 37077 Göttingen, Germany.

[2]Max Planck Institute for Biophysical Chemistry, Research Group Structure and Function of Molecular Machines, Am Fassberg 11, 37077 Göttingen, Germany.

[3]University Medical Center Göttingen, Department of Cellular Biochemistry, Humboldtallee 23, 37073 Göttingen, Germany.

[*]Correspondence to Patrick Cramer (pcramer@mpibpc.mpg.de)

Table of Contents

Supplementary Figure 1 2
Supplementary Figure 2 4
Supplementary Figure 3 6
Supplementary References 7

Supplementary Figure 1

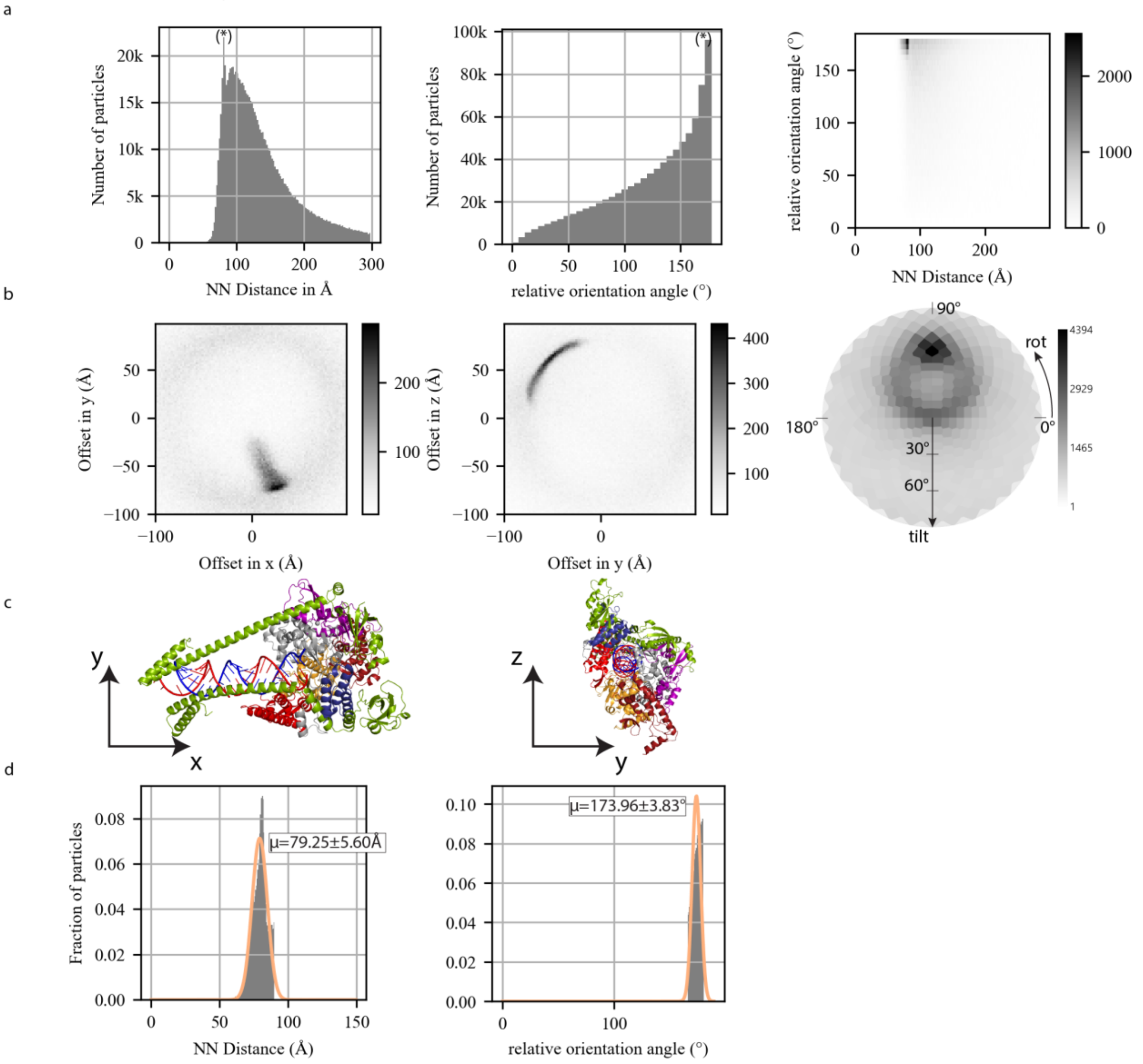

Supplementary Figure 1 Detection of dimeric RdRp particles.

a, Detection of RdRp dimers in cryo-EM data. On the left, nearest neigbor (NN) distances for all N= 902,303 Warp[1]-picked monomeric RdRp particles, with the most significant peak that deviates from the underlying random distribution indicated (*) at around 75 Å. In the middle plot, NN orientations are shown, expressed as a single angle of rotation around the eigenvector of the rotation matrix. A clear peak (*) is visible close to 180°. On the right, a conjoined plot of NN monomer distances and relative orientations, revealing that those nearest neighbors with a distance around 75 Å are also very likely to have relative orientation of >165°. This indicates a correlation between this distance and a defined orientation within dimers.

b, Detailed analysis of our peak distances and relative orientations. In the first two panels, the elements of the vector connecting two NN monomers, expressed in the reference frame of one of the

two monomers, are shown, further indicating a defined co-localization of two monomeric RdRps. In the third panel, a projection of relative orientations on a half sphere are shown. Colors indicate the number of particles in each bin.

c, Monomeric RdRp as previously reported[2] (PDB code 6yyt) with axes indicated that were used for x,y,z offsets in **b**. Color code is the same as in **Supplementary Figure 3.**

d, NN distances and relative orientations of the N= 31,011 monomers that are predicted to be part of dimers and an overlayed Gaussian fit**.**

Supplementary Figure 2

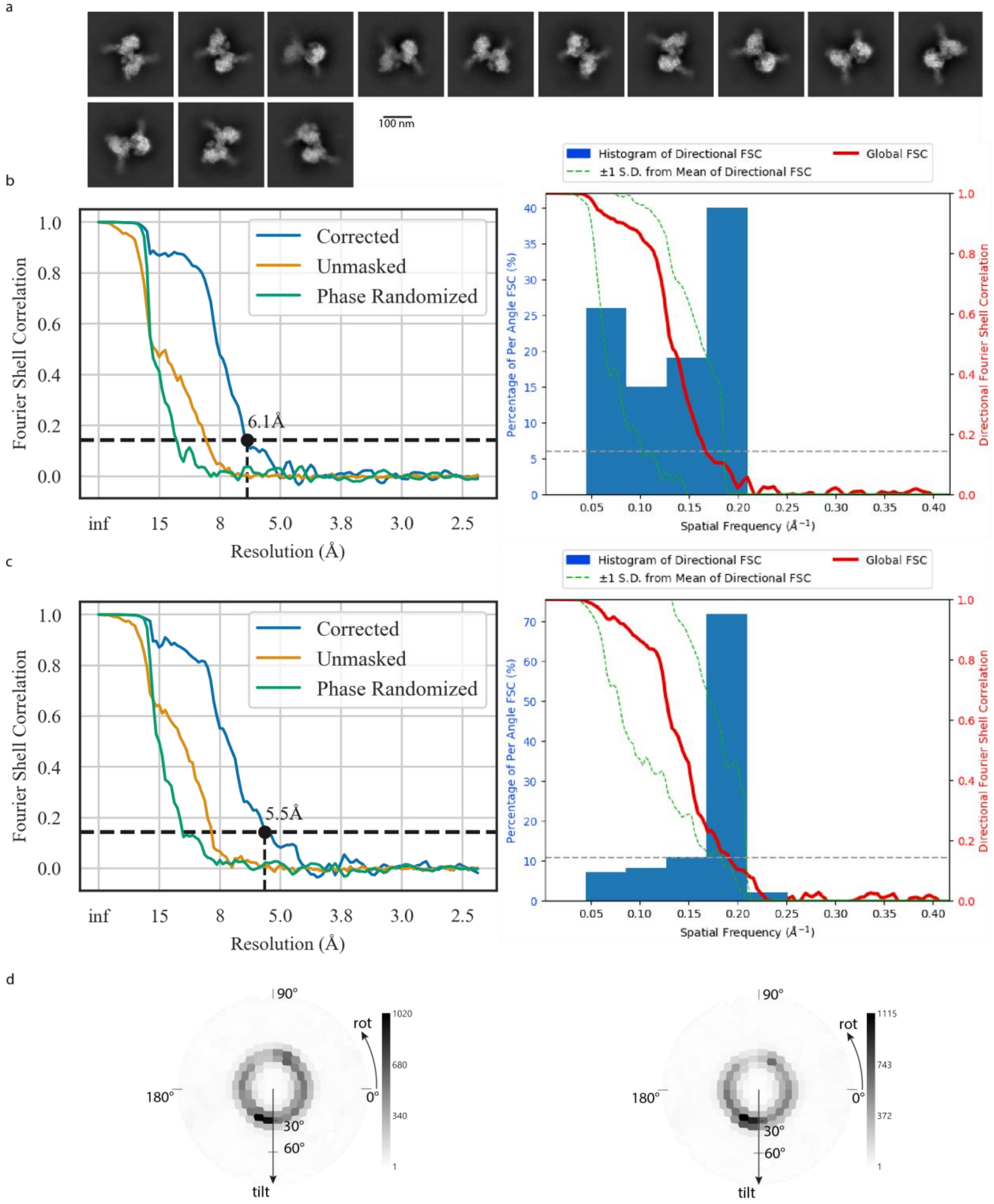

Supplementary Figure 2 Cryo-EM processing of RdRp dimers.

a, Selected 2D class averages from our predicted 78,787 dimeric particles. We chose classes with two clearly defined RdRp monomers and strong RNA signal as an indication for high alignment quality, representing 27,473 particles in total.

b, Fourier shell correlation (FSC) curve for the masked reconstruction of the antiparallel dimer, indicating an average resolution of 6.1 Å. FSC values were calculated through a RELION[3] postprocessing job to obtain values for the phase randomized curve as well. As input, the half maps and refinement mask from cryoSPARC's[4] reconstruction were provided. The second panel contains a histogram of directional FSC values as calculated by the 3DFSC[5] server, indicating significant anisotropy with a sphericity value of 0.807.

c, With C2 symmetry applied, the average resolution of the reconstruction increased to 5.5 Å. The second panel contains a histogram of directional FSC values as calculated by the 3DFSC server, indicating significant anisotropy with a sphericity score of 0.789.

d, Orientation distribution from the reconstruction without (left) and with C2 symmetry alignment (right), showing a strong preferred orientation. Colour bars indicate number of particles in each bin.

Supplementary Figure 3

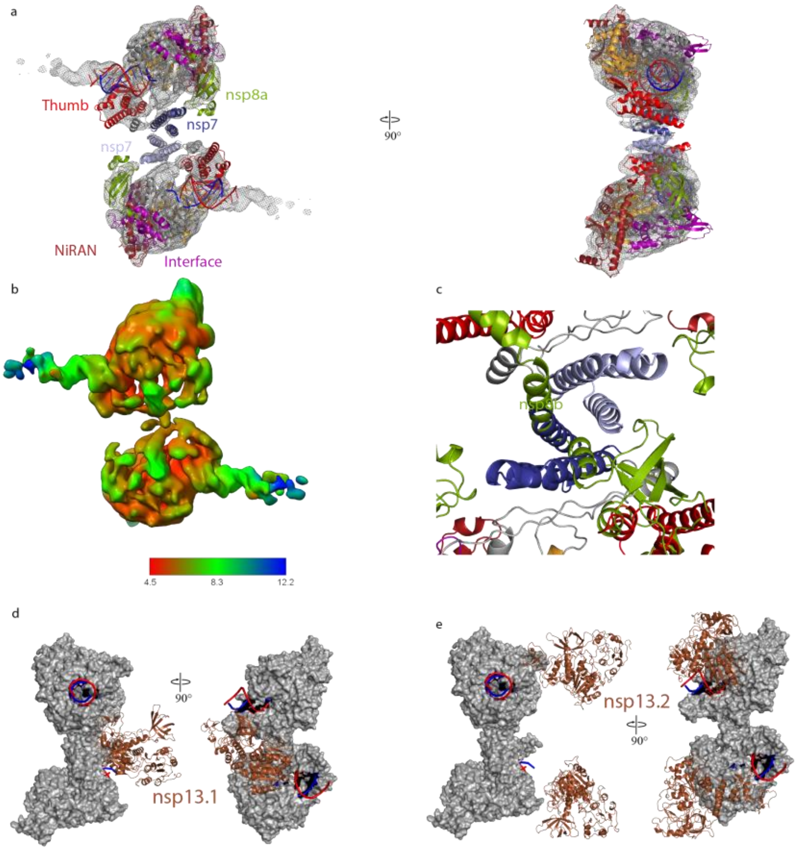

Supplementary Figure 3: Quality of dimeric RdRp structure and structural comparisons.

a, Fit of structural model to cryo-EM density. Density was weak or lacking for the second turn of RNA and the associated nsp8a sliding pole. Views are related by a 90° rotation around the vertical axis.

b, Local resolution estimated using cryoSPARC. Resolution was weak for the second turn of RNA, which was also excluded from our model in **a**.

c, Modeling the second nsp8 copy (nsp8b) onto the top RdRp in the dimer structure resulted in clashes with the nsp7 subunit of the neighboring RdRp on the bottom.

d, Modeling of the nsp13 shows that helicase may accommodate on the RdRp dimeric structure. The nsp13 copy nsp13.1 (chain E in 7CXN[6]) can bind to one of the two monomers (binding to top monomer shown).

e, Modeling **of the** nsp13.2 shows that each of the two RdRp molecules may bind **this copy of nsp13** (chain F in 7CXN[6]) without clashes.

Supplementary References

1. Tegunov, D. & Cramer, P. Real-time cryo-electron microscopy data preprocessing with Warp. *Nat. Methods* **16**, 1146–1152, doi:10.1038/s41592-019-0580-y (2019).

2. Hillen, H. S. *et al.* Structure of replicating SARS-CoV-2 polymerase. *Nature* **584**, 154–156, doi:10.1038/s41586-020-2368-8 (2020).

3. Scheres, S. H. W. RELION: Implementation of a Bayesian approach to cryo-EM structure determination. *J. Struct. Biol.* **180**, 519–530, doi:10.1016/j.jsb.2012.09.006 (2012).

4. Punjani, A., Rubinstein, J. L., Fleet, D. J. & Brubaker, M. A. CryoSPARC: Algorithms for rapid unsupervised cryo-EM structure determination. *Nat. Methods* **14**, 290–296, doi:10.1038/nmeth.4169 (2017).

5. Zi Tan, Y. *et al.* Addressing preferred specimen orientation in single-particle cryo-EMthrough tilting. *Nat. Methods* **14**, 793–796, doi:10.1038/nmeth.4347 (2017).

6. Yan, L. *et al.* Architecture of a SARS-CoV-2 mini replication and transcription complex. *Nat. Commun.* **11**, 3–8, doi:10.1038/s41467-020-19770-1 (2020).
