# Supplementary material for: The structure of a dimeric form of SARS-CoV-2 polymerase
Source: Commun Biol. 2021 Aug 24;4:999. doi: 10.1038/s42003-021-02529-9 (PMC8385044; doi:10.1038/s42003-021-02529-9)
Supplement: Supplementary file 3 — Description of Additional Supplementary Files [file 42003_2021_2529_MOESM3_ESM.pdf]

Description of Additional Supplementary Files

File name: Supplementary Data 1

Description: Source Data for Supplementary Figure 1
